# Incretin receptor agonists rapidly and markedly attenuate multi‐cause insulin resistance in the hippocampal formation from MCI and AD dementia cases

**DOI:** 10.1002/alz.089821

**Published:** 2025-01-09

**Authors:** Konrad Talbot

**Affiliations:** ^1^ Loma Linda University Health, Loma Linda, CA USA

## Abstract

**Background:**

Only about 50% of the variance in cognitive decline occurring during Alzheimer’s pathogenesis is attributable to standard AD biomarkers (cerebrocortical Aβ, pathological tau, and atrophy) (Tosun et al., Alzheimer’s Dement. 18: 1370, 2022). Other factors contributing to such decline include brain insulin resistance (BIR). Levels of its biomarker, pathological IRS‐1 pS616 (insulin receptor substrate‐1 phosphorylated at S616), accounts for as much as 47% of the variance in episodic memory scores in an elderly population we studied (Talbot et al., JCI 122: 1316, 2012). To better understand BIR in Alzheimer’s disease (AD), its proximal causes, and if incretin receptor agonists (IRAs) can reduce BIR, we extended our earlier ex vivo work on the hippocampal formation (HF) of non‐cognitively impaired (NCI) and AD dementia (ADd) cases to non‐amnestic MCI (naMCI) and amnestic MCI (aMCI) cases

**Method:**

Diverse brain banks donated fresh frozen HF tissue from NCI, naMCI, aMCI, and ADd cases (n = 10 per diagnostic group) who were age‐ and ‐sex matched whites with no history of diabetes and low postmortem intervals (m ± SD = 6.75 ± 2.2 h). Using our ex vivo stimulation protocol (Talbot et al., 2012), tissue sections were tested for insulin‐induced phosphorylation of insulin signaling and regulating enzymes after 30 min incubations in 0, 1, or 10 nM insulin with or without 30 min preincubation in 100 nM of liraglutide activating GLP‐1 (glucagon‐like peptide‐1) receptors, [D‐Ala^2^]‐GIP activating GIP (gastric inhibitory peptide) receptors, or Peptide 19, a dual IRA activating both GLP‐1 and GIP receptors. Statistical significance was defined as p < 0.05.

**Result:**

HF responsiveness to 1 and 10 nM insulin was significantly reduced in both the MCI and ADd samples. Pretreatment with each of the IRAs tested virtually restored normal insulin responsiveness in both MCI groups even though the proximal cause of insulin resistance in naMCI differed from aMCI and ADd. Only the dual IRA, however, significantly increased insulin responsiveness in ADd samples.

**Conclusion:**

IRAs reaching brain neurons can virtually eliminate hippocampal insulin resistance in naMCI and aMCI cases, but significantly reducing such resistance in ADd cases appears to require a dual IRA.

